# Evidence for Extracellular Superoxide Dismutase (SOD3), Glutathione and Redox Dynamics in Amniotic Fluid Throughout Gestation

**DOI:** 10.3390/children12081086

**Published:** 2025-08-19

**Authors:** Leah Knieps, Ebru Aileen Alsat, Tamene Melaku, Andreas Mueller, Soyhan Bagci

**Affiliations:** Neonatology and Pediatric Intensive Care, Children’s Hospital, University of Bonn, 53127 Bonn, Germany; leah.knieps@gmail.com (L.K.); ebru_aileen.alsat@ukbonn.de (E.A.A.); tamene.melaku@ukbonn.de (T.M.); a.mueller@ukbonn.de (A.M.)

**Keywords:** amniotic fluid, human milk, foetus, gastrointestinal development, redox signalling, superoxide dismutase 1, superoxide dismutase 3

## Abstract

**Highlights:**

**What are the main findings?**

- Our study provides novel insights by characterising the antioxidant profiles of amniotic fluid (AF), with a specific focus on superoxide dismutase isoforms (SOD1 and SOD3), glutathione (GSH), and 8-OHdG.
- AF provides a redox-regulated microenvironment during foetal development of the gastrointestinal tract (GIT).
- Antioxidant environment of AF is dynamic, undergoing substantial modulation of the oxidative–antioxidative balance throughout gestation.

**What is the implication of the main finding?**

- Gestational age-specific components of AF, including enzymatic antioxidants such as SOD1, SOD3, and GSH, should be considered in the development of targeted nutritional and pharmacological interventions, particularly for vulnerable populations such as preterm infants and those with congenital gastrointestinal anomalies.

**Abstract:**

Introduction: Amniotic fluid (AF) plays a pivotal role in foetal gastrointestinal development by delivering bioactive factors that support intestinal maturation. However, the redox environment of AF and its potential contribution to foetal intestinal homeostasis remain insufficiently characterised. This study aimed to quantify key antioxidant markers—superoxide dismutase isoforms (SOD1, SOD3), glutathione (GSH), and the oxidative DNA damage marker 8-hydroxy-2-deoxyguanosine (8-OHdG)—in AF across gestational ages and compare them with those in human milk (HM). Methods: AF samples (*n* = 60) were collected from pregnancies between 15 and 40 weeks of gestation, grouped into preterm (<37 weeks) and term (≥37 weeks). SOD1, SOD3, GSH, and 8-OHdG concentrations were quantified using ELISA. HM samples (*n* = 45) were similarly analysed. Results: SOD1 and SOD3 in AF concentrations decreased significantly with gestational age (GA) (*p* < 0.001), while 8-OHdG levels increased (*p* < 0.001). SOD3 showed a negative correlation with 8-OHdG (*p* = 0.004). HM contained significantly higher levels of both SOD isoforms compared to AF (AF vs. HM: 35.6 (1.9–172.3) vs. 267.9 (54.6–843.8), *p* < 0.001 for SOD1 and 1.2 ng/mL (0.1–26.5) vs. 5.5 ng/mL (0.1–300.0), *p* < 0.001 for SOD3), regardless of GA. Conclusions: Our findings highlight the dynamic nature of the redox environment in AF and its potential importance for foetal GIT development. The disruption of redox balance by preterm birth or inadequate AF intake during foetal life may have long-term consequences for intestinal development and function. These insights provide a foundation for future clinical studies aimed at enhancing neonatal feeding regimens, particularly for preterm infants and those with congenital gastrointestinal disorders.

## 1. Introduction

The development and maturation of the gastrointestinal tract (GIT) is a dynamic, multi-stage process that begins in utero and continues into early postnatal life. After mid-gestation, the intestinal epithelium undergoes structural and functional maturation, characterised by coordinated cellular proliferation, differentiation, and apoptosis to establish the crypt–villus axis and prepare the gut for the transition to extra-uterine nutrition [1]. This process is governed by a complex interplay of genetic programming, endocrine signals, and paracrine factors derived from the surrounding mesenchyme and amniotic fluid (AF) [1,2]. Among the key regulators of these processes is redox signalling, wherein reactive oxygen species (ROS) serve as critical signalling molecules that play a key role in cellular processes such as cell proliferation, differentiation, and apoptosis [3,4]. It is established that both cellular and luminal redox environments are crucial in preserving cellular integrity and maintaining the overall redox homeostasis of tissues [4,5]. The developmental regulation of intestinal growth and adaptation relies on an appropriate balance between the antioxidant system and ROS. Investigating this relationship in vivo presents challenges due to the dual role of ROS: at physiological levels, ROS serve as signalling molecules, whereas an imbalance between ROS production and antioxidant defence—known as oxidative stress—can disrupt these signalling pathways, impair tight junction formation, and promote inflammatory responses, thereby compromising intestinal barrier function [3]. This redox-dependent regulation may be relevant in the neonatal gut, especially in preterm infants, where varying oxygen levels, microbial colonisation, and nutritional changes modulate ROS levels and redox-sensitive pathways that shape postnatal intestinal adaptation.

Recent research has highlighted that foetal gastrointestinal development, growth, and maturation are critically dependent on the ingestion of AF [2,6,7]. By around the tenth week of gestation, the foetus begins to swallow AF, and this process increases steadily so that near term, the daily intake reaches approximately 700 mL [2]. AF is a complex biological fluid containing a diverse array of components, including proteins, hormones, prostaglandins, catecholamines, glucose, electrolytes, enzymes, and growth factors—all of which directly interact with the intestinal epithelium to help prepare the gut for an extra-uterine environment [7,8]. In preterm infants, however, early delivery interrupts this natural process, terminating the normal period of AF ingestion and intestinal absorption that generally occurs in the third trimester. Similarly, in-term infants with certain congenital anomalies—such as congenital diaphragmatic hernia, oesophageal atresia, or intestinal atresia—the anatomical abnormalities physically obstruct the normal flow of AF into the gut. This disruption in AF uptake can compromise gut maturation, alter mucosal development, and increase the risk of postnatal feeding difficulties, as well as complications such as necrotising enterocolitis. A thorough understanding of the composition and functions of AF is therefore essential for enhancing nutritional strategies and reducing both short- and long-term morbidity in critically ill preterm and term newborn infants.

Despite our growing understanding of its many constituents, much less is known about the redox environment in AF. Superoxide dismutase (SOD) is a key enzymatic antioxidant that catalyses the dismutation of superoxide anions (O_2_^−^) into molecular oxygen (O_2_) and hydrogen peroxide (H_2_O_2_), thereby mitigating oxidative stress [9]. In humans, SOD exists in three isoforms—SOD1, SOD2, and SOD3—each differing in cellular localisation and metal cofactor requirements [10]. To date, studies measuring SOD activity in AF have not distinguished between SOD isoforms [11,12,13]. SOD3, the principal extracellular isoform, is present in various tissues and fluids, where it exerts distinct immunomodulatory and protective functions, including anti-inflammatory and anti-angiogenic effects, as well as the regulation of nitric oxide (NO)-mediated signalling [14,15]. Glutathione (GSH) is the most abundant intracellular thiol in intestinal epithelial cells and plays a central role in maintaining redox homeostasis, detoxifying ROS, and supporting epithelial barrier integrity [16]. A deeper exploration of the antioxidative system in AF could offer valuable insights for improving postnatal outcomes, particularly in preterm newborns.

Gaining a deeper understanding of the antioxidative system in AF could be key to improving postnatal outcomes in preterm and term newborn infants by supporting optimal gut development and overall health. Our study aimed to provide novel insights into the characterisation of the antioxidant profiles of AF, with a specific focus on superoxide dismutase isoforms (SOD and SOD3) and glutathione (GSH), which are critical for stem/progenitor cell proliferation, differentiation, and barrier function in the developing gut [15,17]. Moreover, we analysed 8-hydroxy-2-deoxyguanosine (8-OHdG), a marker of oxidative DNA damage [18].

## 2. Materials and Methods

### 2.1. Subjects

Sixty pregnant women were included in the study. Inclusion criteria were: availability of GA determination by both last menstrual period and first- or second-trimester ultrasonographic foetal biometry, and clinical indication for amniocentesis or cesarean delivery. Exclusion criteria were the presence of maternal or foetal conditions that could affect AF composition, including chorioamnionitis, premature rupture of membranes, pre-eclampsia/eclampsia, gestational diabetes requiring insulin, and bilateral renal agenesis. Women with any systemic inflammatory disease or chronic medication use known to influence oxidative stress markers were also excluded.

GA was evaluated using the last menstrual period and ultrasonographic foetal biometry; discrepancies > 7 days prompted correction using biometric dating. Participants were grouped according to GA into Group 1 (<37 completed weeks; *n* = 36) and Group 2 (≥37 weeks; *n* = 24), with the cut-off reflecting the World Health Organization’s definition of term pregnancy. In accordance with the Declaration of Helsinki, the study was approved by the Ethics Committee of the University of Bonn (503/20, approved on 23 November 2020). An informed consent form for participation was distributed to all participants and signed.

### 2.2. AF and Human Milk (HM) Collection Procedure

AF samples were obtained under sterile conditions either during clinically indicated amniocentesis or at the time of elective or emergency cesarean section performed under general anaesthesia. AF samples were obtained by needle aspiration that was visually free of bloody contamination. Within 30 min of collection, AF was centrifuged at 3000× *g* for 10 min at 4 °C to remove cellular debris. The supernatant was aliquoted into low-protein-binding tubes in 0.5–1 mL volumes to minimise repeated freeze–thaw cycles and stored at –80 °C until analysis. All samples underwent only a single freeze–thaw cycle prior to biochemical assays.

For HM samples, the mothers were enrolled in the study between the 5th and 10th day postpartum. All women were asked to fill 2 mL of breast milk in tubes after expressing and to freeze them at −20 °C. The women carried the tubes to us, and they were then frozen at −80 °C until analysis. Prior to analysis, the samples were thawed and centrifuged at 3000× *g* for 10 min at 4 °C. The supernatant was pipetted and used for analysis.

### 2.3. Analytical Methods

The concentrations of SOD1, SOD3, GSH, and 8-OHdG in AF were determined by the enzyme-linked immunosorbent assay (ELISA) method (SOD1: Cat. No.: BMS222, SOD3: Cat. No.:EH432RB, Thermofisher, Life Technologies GmbH, Darmstadt, Germany; GSH: Cat. No: KTE62857, Abbkine, Atlanta, GA, USA, and 8-OHdG: Cat. No. KOG-200S/E, Japan Institute for the Control of Aging, Shizuoka, Japan). Assays were performed strictly according to the manufacturers’ instructions, with all standards and samples run in duplicate. Calibration curves were generated for each plate, and both positive and negative controls provided by the manufacturer were included to verify assay performance. Intra-assay coefficients of variation (CV) were 5.1% for SOD1, <10.0% for SOD3, <9% for GSH, and 4.0% for 8-OHdG; inter-assay CVs were 5.8%, <12.0%, <11%, and 5.4%, respectively. The limits of detection (LOD) and quantification (LOQ) were as specified by the kit manufacturers and verified in our laboratory using serial dilution of pooled samples.

### 2.4. Statistical Analyses

The statistical analyses were performed using SPSS 27.0 (SPSS Inc., Chicago, IL, USA). All data are presented as median (interquartile range (IQR)). The normality of the variables was tested using the Kolmogorov–Smirnov and Shapiro–Wilk tests. Because SOD1, SOD3, and 8-OHdG levels were not normally distributed, we used the two-tailed non-parametric Mann–Whitney U-test for the group comparison. Spearman correlation analysis was used to analyse associations among GA, SOD1, SOD3, GSH, and 8-OHdG levels. Where multiple comparisons were made, the Bonferroni correction was applied to adjust *p*-values. According to GA, the samples were divided into two categories: Group 1 (*n* = 36) includes AF samples obtained before 37 weeks of gestation, and Group 2 (*n* = 24) includes AF samples obtained after 37 weeks of gestation. For each statistical test, exact sample sizes (*n*) are reported in the tables and figure legends. A *p*-value < 0.05 (two-tailed) was considered statistically significant

## 3. Results

**Figure 1:**
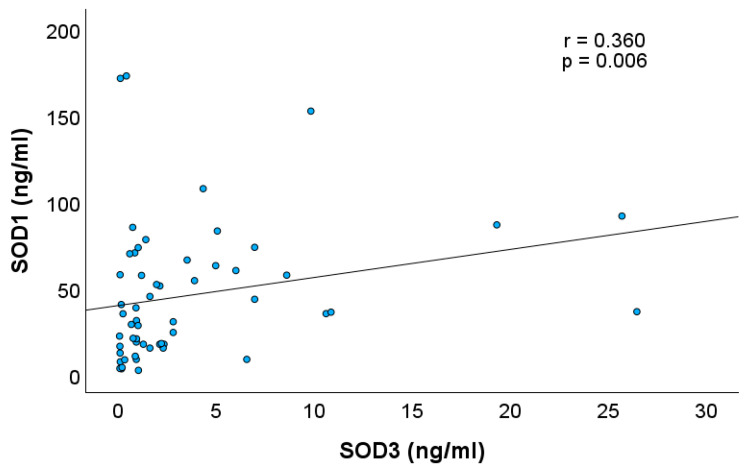
Correlation between SOD1 and SOD3 concentrations.

**Figure 2:**
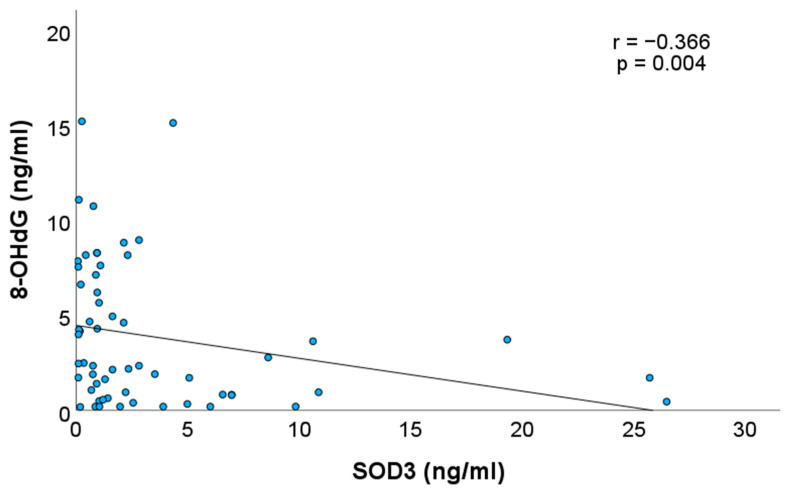
Correlation between 8-OHdG and SOD3 concentrations.

Table 1 demonstrates the concentrations of SOD1, SOD3, GSH, and 8-OhdG in AF. The concentration of SOD1 was significantly higher than that of SOD3 (*p* < 0.001). SOD1 and SOD3 concentrations in AF showed a significant negative correlation with GA, indicating a progressive decrease in their levels as pregnancy progressed (Spearman correlation coefficient, r = −0.450, *p* < 0.001, and r = −0.429, *p* < 0.001, respectively). GSH showed no correlation with GA (*p* = 0.165). Furthermore, a positive correlation was observed between SOD1 and SOD3 concentrations (r = 0.360, *p* = 0.006) (Figure 1). The concentration of 8-OHdG in AF increased as pregnancy advanced (r = 0.636, *p* < 0.001). There was also a significant negative correlation between 8-OHdG and SOD3 (r = −0.366, *p* = 0.004) (Figure 2), while SOD1 and GSH showed no correlation with 8-OHdG level (r = −0.214, *p* = 0.11 and −0.252, *p* = 0.056, respectively).

Table 2 presents the concentrations of SOD1 and SOD3 in human milk (HM) samples from 45 women. SOD3 levels showed marked interindividual variability, ranging from 0.1 to 300 ng/mL. SOD1 concentrations were significantly higher than those of SOD3 (*p* < 0.001). Both SOD1 and SOD3 in HM were positively correlated with GA (r = 0.224, *p* = 0.034, and r = 0.248, *p* = 0.026, respectively). However, no significant differences were observed between preterm and term samples (*p* = 0.390 and *p* = 0.344). A significant positive correlation was also found between SOD1 and SOD3 concentrations (r = 0.351, *p* = 0.001). In comparison to AF, SOD1 and SOD3 levels were significantly higher in both preterm (*p* < 0.001 and *p* = 0.008) and term HM (*p* < 0.001).

## 4. Discussion

The role of antioxidants and redox signalling in intestinal development is a critical and complex aspect of gastrointestinal physiology, particularly in the context of early life, including foetal and neonatal gastrointestinal development. The findings of this study support the hypothesis that AF provides a redox-regulated microenvironment. We observed a decline in the concentrations of both SOD isoforms with increasing GA, accompanied by an increase in the oxidative DNA damage marker 8-OHdG. These results suggest that the antioxidant environment of AF is dynamic, undergoing substantial modulation of the oxidative–antioxidative balance throughout gestation. This modulation may be related to developmental regulation, tissue maturation, the evolving demands of the developing foetus, or changes in the composition of the AF source.

It is well established that the redox environment plays a crucial role in preserving cellular integrity and maintaining overall tissue redox homeostasis in the intestine, whose high regenerative capacity is primarily attributed to Intestinal Stem Cells (ISCs) [5]. Intracellular and luminal redox environments must be tightly regulated; too much or too little ROS could inhibit cell growth. When the intestinal redox environment is disrupted, for instance, by sustained oxidative stress or an imbalance in antioxidant defences, the resulting redox dysregulation can compromise cell fate decisions and lead to tissue dysfunction, chronic inflammation, or programmed cell death [19]. Our study offers novel insights into the antioxidant profiles of AF, with a specific focus on superoxide dismutase isoforms (SOD1 and SOD3), GSH, and 8-OHdG. The presence of SOD1 in AF has been previously reported, yet our study is the first to quantify SOD3 in AF [11]. SOD3 is a key antioxidant in the extracellular matrix, protecting against oxidative stress and thereby maintaining tissue health. It also regulates nitric oxide signalling, which is essential for vascular and tissue development [14,20]. Moreover, SOD3 can significantly mitigate enteritis symptoms by preserving the integrity of epithelial junctions and regulating inflammatory and oxidative stress responses [15]. In addition to SOD3, we identified GSH, which can be transported intact into the small intestine in vivo, may contribute to maintaining mucosal thiol balance [16,21]. Moreover, Kovacs-Nolan et al. [22] demonstrated in their in vitro and ex vivo uptake study of GSH that intact GSH can be transported across both the basolateral and apical surfaces of intestinal epithelial cells. The studies suggest that luminal GSH could help protect the gastrointestinal epithelium from inflammatory, ischemic, and oxidative injury [16,21,23]. Taken together, the presence of SOD3 and GSH in AF therefore raises the possibility that they contribute to feotal gastrointestinal development by supporting ISC function, maintaining redox balance, and safeguarding epithelial integrity during gut maturation. Importantly, while these patterns suggest physiological roles for SOD3 and GSH, causal relationships remain to be demonstrated. Preclinical studies—such as functional assays on intestinal epithelial barrier integrity, antioxidant supplementation trials in animal models, and the use of organoid systems—are needed to clarify mechanisms and assess whether modulating antioxidant exposure could influence developmental outcomes. For preterm neonates, who may experience an abrupt loss of SOD3-rich AF, such studies would help determine whether supplementation strategies can safely replicate aspects of the intrauterine redox environment.

The positive correlation between SOD1 and SOD3 concentrations suggests coordinated regulation or a shared source, meriting further investigation into their developmental control and interaction with other redox-sensitive signalling networks [24]. The significant negative correlation between GA and concentrations of SOD1 and SOD3 suggests that the early intrauterine environment is more reliant on antioxidant protection, which diminishes as structural and functional maturation of the foetal GIT progresses. SOD3 concentrations were low overall and highly variable between individuals. Given its extracellular localisation and role in NO signalling and vascular integrity [20], a late gestational decline in SOD3 may reflect a physiological shift toward a more pro-oxidant state, potentially facilitating terminal differentiation and immune priming. The gestational increase in 8-OHdG could represent both a rise in oxidative stress and enhanced ROS-mediated signalling necessary for epithelial differentiation, morphogenesis, and immune maturation. This finding is consistent with previous studies, which have demonstrated that redox signalling plays a crucial role in orchestrating epithelial proliferation, differentiation, and apoptosis [3,4]. The observed correlations between SOD isoforms, 8-OHdG, and GA reinforce the concept of a dynamic yet balanced antioxidant network in the foetal compartment. Moreover, the negative correlation between 8-OHdG and SOD3—but not SOD1—may point to the unique extracellular role of SOD3 in mitigating oxidative DNA damage, potentially in the foetal gut mucosa directly exposed to swallowed AF. This may serve to protect against basal or environmentally induced oxidative stress, while allowing controlled ROS signalling necessary for developmental cues. However, in preterm neonates lacking this gradual transition, the premature loss of SOD3-rich AF may exacerbate redox vulnerability in the immature gut. From a translational perspective, our findings support the notion that gestational age-specific components of AF, including enzymatic antioxidants such as SOD1 and SOD3, should be considered in the development of targeted nutritional and pharmacological interventions for at-risk neonates.

Many aspects of how redox signalling influences foetal intestinal development remain poorly understood. In particular, it is unclear how fluctuations in ROS and the activity of antioxidant systems influence epithelial cell differentiation, proliferation, and maturation during the early neonatal period, especially in preterm infants. Clarifying this mechanism could help reduce morbidity and mortality in two vulnerable neonatal populations: preterm infants and those with congenital anomalies that prevent swallowing of amniotic fluid, such as congenital diaphragmatic hernia (CDH), oesophageal or intestinal atresia.

In preterm infants, the intestinal redox environment rapidly shifts from a hypoxic, reducing intrauterine state to a more oxidative postnatal state due to oxygen exposure, microbial colonisation, and underdeveloped antioxidant defences—factors that collectively increase oxidative stress. Our comparative analyses show that SOD1 and SOD3 levels are significantly higher in HM than in AF, especially during early gestation. However, the biological impact of this abrupt redox transition, compounded by the shift from amniotic fluid to human milk or formula feeding, remains insufficiently studied. Another critical concern is the gastrointestinal development of foetuses with CDH or intestinal atresia, who experience a complete or near-complete lack of AF ingestion during gestation. These infants often present with severe gastrointestinal immaturity, resulting in delayed initiation of enteral feeding, extended reliance on parenteral nutrition, and a high incidence of complications such as intestinal ischemia, feeding intolerance, dysphagia, growth failure, and gastro-oesophageal reflux, with lasting effects on nutrition and quality of life [25]. In CDH, delayed enteral nutrition—independent of pulmonary pathology—has emerged as a major contributor to prolonged hospitalisation and reduced postnatal outcomes. Given the shared pathophysiology of AF deprivation, early postnatal enteral nutrition strategies should aim to mimic the trophic, protective, and maturational roles of swallowed AF. These insights also underscore the importance of investigating whether exogenous antioxidant enzyme supplementation—via fortified milk, parenteral solutions, or pharmacological agents—can replicate the protective effects of AF and enhance outcomes in neonatal care. To accelerate gastrointestinal maturation in these high-risk neonates, postnatal enteral nutrition with AF may serve as a physiologically tailored strategy to replicate the trophic and redox-regulatory roles of prenatal AF ingestion. Further preclinical and translational studies are needed to evaluate its feasibility and safety.

This study has several limitations. First, the sample size, although adequate for statistical analysis, limits the generalisability of the findings and warrants validation in larger cohorts. Second, we did not account for other antioxidants present in AF, such as catalase, which could provide a more comprehensive view of the antioxidant landscape. We did not analyse glutathione peroxidase in this study, as our previous work showed that glutathione peroxidase concentrations in AF were very low or below the limit of detection. Third, the lack of functional assays limits our ability to directly link enzyme concentrations to biological effects on intestinal development. Future studies should investigate the temporal expression and function of these enzymes in foetal intestinal tissue and assess whether exogenous supplementation of antioxidants in preterm feeding regimens can recapitulate intrauterine conditions and improve outcomes.

## 5. Conclusions

Our findings highlight the dynamic nature of the antioxidant environment in AF and its potential importance for foetal GIT development. The gestational decline in SOD1 and SOD3 levels, coupled with an increase in oxidative DNA damage, underscores the importance of a tightly regulated redox balance that may be crucial for proper intestinal maturation. The disruption of this balance by preterm birth or lack of AF intake during foetal life, followed by formula feeding, may have long-term consequences for intestinal integrity and function. These insights provide a foundation for future clinical studies aimed at enhancing neonatal feeding regimens, particularly for preterm infants and those with congenital gastrointestinal disorders. While mimicking the trophic and protective effects of swallowed AF is an attractive concept, the safety, feasibility, and efficacy of interventions such as AF-derived nutritional supplementation must be rigorously evaluated in preclinical models before clinical application.

## Figures and Tables

**Table 1:** SOD1, SOD3, GSH, and 8-OHdG concentration in amniotic fluid.

	All Samples (*n* = 60)	Group 1 (*n* = 36)	Group 2 (*n* = 24)	*p*-Value
Gestational Age (GA) (weeks)	35.2 (15.0–40.0)	22.4 (15.0–36.7)	38.7 (37.1–40.0)	<0.001
SOD1 (ng/mL)	35.6 (1.9–172.3)	51.6 (3.6–170.9)	19.2 (1.9–172.3)	0.002
SOD3 (ng/mL)	1.2 (0.1–26.5)	2.1 (0.1–26.5)	0.8 (0.1–4.4)	<0.001
GSH (ng/L)	71.1 (50.4–103.9)	74.8 (55.2–100.9)	66.0 (50.4–103.9)	0.167
8-OHdG (ng/mL)	2.4 (0.2–15.2)	1.5 (0.2–7.6)	6.0 (0.2–15.2)	<0.001

The data are expressed as the median (IQR). SOD: superoxide dismutase; 8-OHdG: 8-hydroxy-2-deoxyguanosine. Group 1: <37 weeks of gestation; Group 2: ≥37 weeks of gestation.

**Table 2:** SOD1 and SOD3 concentration and GA of the analysed human milk samples.

	All Samples (*n* = 90)	Group 1 (*n* = 65)	Group 2 (*n* = 25)	*p*-Value
Gestational Age (weeks)	34.2 (25.6–42.1)	32.0 (25.6–36.7)	39.6 (37.6–39.0)	<0.001
SOD1 (ng/mL)	267.9 (54.6–843.8)	252.2 (54.6–843.8)	316.8 (116.0–661.2)	0.390
SOD3 (ng/mL)	5.5 (0.1–300.0)	4.7 (0.1–300.0)	9.1 (0.1–292.5)	0.344

The data are expressed as the median (Range). SOD: superoxide dismutase; Group 1: <37 weeks of gestation; Group 2: ≥37 weeks of gestation.

## Data Availability

The original contributions presented in this study are included in the article. Further inquiries can be directed to the corresponding author.

## Abbreviations

The following abbreviations are used in this manuscript:

AF	Amniotic fluid
CDH	Congenital diaphragmatic hernia
GA	Gestational age
GIT	Gastrointestinal tract
HM	Human milk
ISCs	Intestinal Stem Cells
H_2_O_2_	Hydrogen peroxide
NO	Nitric oxide
O_2_	Molecular oxygen
O_2_^−^	Superoxide anions
ROS	Reactive oxygen species
SOD	Superoxide dismutase
SOD1	Intracellular Cu/Zn superoxide dismutase
SOD3	Extracellular Cu/Zn superoxide dismutase
8-OHdG	8-hydroxy-2-deoxyguanosine

## References

[1] Commare C.E., Tappenden K.A. (2017). Development of the Infant Intestine: Implications for Nutrition Support. Nutr. Clin. Pract..

[2] Bagci S., Brosens E., Tibboel D., de Klein A., Ijsselstijn H., Wijers C.H.W., Roeleveld N., de Blaauw I., Broens P.M., van Rooij I.A.L.M. (2016). More than fetal urine: Enteral uptake of amniotic fluid as a major predictor for fetal growth during late gestation. Eur. J. Pediatr..

[3] Morris O., Jasper H. (2021). Reactive Oxygen Species in intestinal stem cell metabolism, fate and function. Free Radic. Biol. Med..

[4] Nath A., Chakrabarti P., Sen S., Barui A. (2022). Reactive Oxygen Species in Modulating Intestinal Stem Cell Dynamics and Function. Stem Cell Rev. Rep..

[5] Circu M.L., Aw T.Y. (2012). Intestinal redox biology and oxidative stress. Semin. Cell Dev. Biol..

[6] Buchanan K.L., Bohórquez D.V. (2018). You Are What You (First) Eat. Front. Hum. Neurosci..

[7] Underwood M.A., Gilbert W.M., Sherman M.P., Underwood M.A., Gilbert W.M., Sherman M.P. (2005). Amniotic Fluid: Not Just Fetal Urine Anymore. J. Perinatol. Off. J. Calif. Perinat. Assoc..

[8] Tong X.L., Wang L., Gao T.B., Qin Y.G., Qi Y.Q., Xu Y.P. (2009). Potential Function of Amniotic Fluid in Fetal Development—Novel Insights by Comparing the Composition of Human Amniotic Fluid with Umbilical Cord and Maternal Serum at Mid and Late Gestation. J. Chin. Med. Assoc..

[9] Rosa A.C., Corsi D., Cavi N., Bruni N., Dosio F. (2021). Superoxide Dismutase Administration: A Review of Proposed Human Uses. Molecules.

[10] Zelko I.N., Mariani T.J., Folz R.J. (2003). Superoxide dismutase multigene family: A comparison of the CuZn-SOD (SOD1), Mn-SOD (SOD2), and EC-SOD (SOD3) gene structures, evolution, and expression. Economica.

[11] Bagci S., Katzer D., Altuntas Ö., Alsat E.A., Berg C., Rebeggiani L., Bartmann P., Müller A. (2022). The fetal gastrointestinal tract is exposed to melatonin and superoxide dismutase rich amniotic fluid throughout prenatal development. J. Clin. Biochem. Nutr..

[12] Herway C., Kanninen T., Witkin S.S., Saade G., Fortunato S.J., Menon R. (2013). Ethnic disparity in amniotic fluid levels of hyaluronan, histone H2B and superoxide dismutase in spontaneous preterm birth. J. Perinat. Med..

[13] Bogavac M., Lakic N., Simin N., Nikolic A., Sudji J., Bozin B. (2012). Biomarkers of oxidative stress in amniotic fluid and complications in pregnancy. J. Matern. Fetal Neonatal Med..

[14] Sah S.K., Agrahari G., Kim T.-Y. (2020). Insights into superoxide dismutase 3 in regulating biological and functional properties of mesenchymal stem cells. Cell Biosci..

[15] Tak L.-J., Kim H.-Y., Ham W.-K., Agrahari G., Seo Y., Yang J.W., An E.-J., Bang C.H., Lee M.J., Kim H.-S. (2021). Superoxide Dismutase 3-Transduced Mesenchymal Stem Cells Preserve Epithelial Tight Junction Barrier in Murine Colitis and Attenuate Inflammatory Damage in Epithelial Organoids. Int. J. Mol. Sci..

[16] Ren H., Meng Q., Yepuri N., Du X., Sarpong J.O., Cooney R.N. (2018). Protective effects of glutathione on oxidative injury induced by hydrogen peroxide in intestinal epithelial cells. J. Surg. Res..

[17] Wang Y., Branicky R., Noë A., Hekimi S. (2018). Superoxide dismutases: Dual roles in controlling ROS damage and regulating ROS signaling. J. Cell Biol..

[18] Graille M., Wild P., Sauvain J.-J., Hemmendinger M., Guseva Canu I., Hopf N.B. (2020). Urinary 8-OHdG as a Biomarker for Oxidative Stress: A Systematic Literature Review and Meta-Analysis. Int. J. Mol. Sci..

[19] Lin P.-Y., Stern A., Peng H.-H., Chen J.-H., Yang H.-C. (2022). Redox and Metabolic Regulation of Intestinal Barrier Function and Associated Disorders. Int. J. Mol. Sci..

[20] Nguyen N.H., Tran G.-B., Nguyen C.T. (2020). Anti-oxidative effects of superoxide dismutase 3 on inflammatory diseases. J. Mol. Med..

[21] Aw T.Y. (2005). Intestinal glutathione: Determinant of mucosal peroxide transport, metabolism, and oxidative susceptibility. Toxicol. Appl. Pharmacol..

[22] Kovacs-Nolan J., Rupa P., Matsui T., Tanaka M., Konishi T., Sauchi Y., Sato K., Ono S., Mine Y. (2014). In vitro and ex vivo uptake of glutathione (GSH) across the intestinal epithelium and fate of oral GSH after in vivo supplementation. J. Agric. Food Chem..

[23] Mårtensson J., Jain A., Meister A. (1990). Glutathione is required for intestinal function. Proc. Natl. Acad. Sci. USA.

[24] Lewandowski Ł., Kepinska M., Milnerowicz H. (2020). Alterations in Concentration/Activity of Superoxide Dismutases in Context of Obesity and Selected Single Nucleotide Polymorphisms in Genes: SOD1, SOD2, SOD3. Int. J. Mol. Sci..

[25] Leyens J., Bo B., Heydweiller A., Schaible T., Boettcher M., Schroeder L., Mueller A., Kipfmueller F. (2024). Parents-reported nutrition and feeding difficulties in infants with congenital diaphragmatic hernia after hospital discharge. Early Hum. Dev..

